# CH25H Promotes Autophagy and Regulates the Malignant Progression of Laryngeal Squamous Cell Carcinoma Through the PI3K‐AKT Pathway

**DOI:** 10.1002/cam4.70312

**Published:** 2024-10-21

**Authors:** Zhenfei Xiang, Senquan Yu, Yuxin Xu, Huacai Xiong, Danfei Hu, Qun Li, Zhenhua Wu

**Affiliations:** ^1^ Department of Radiation Oncology Ningbo Medical Center Lihuili Hospital, Ningbo University Ningbo Zhejiang China; ^2^ Department of Oncology The Second Affiliated Hospital of Zhejiang Chinese Medical University Hangzhou Zhejiang China; ^3^ Department of Otolaryngology, Head and Neck Surgery Ningbo Medical Center Lihuili Hospital, Ningbo University Ningbo Zhejiang China

**Keywords:** autophagy, bioinformatics analysis, CH25H, laryngeal squamous cell carcinoma, PI3K‐AKT pathway

## Abstract

**Background:**

Laryngeal squamous cell carcinoma (LSCC) is a type of cancer of the respiratory tract that often presents with subtle symptoms at the early stage and is susceptible to recurrence and metastasis.

**Materials and Methods:**

To find out key regulatory genes involved in LSCC development, we downloaded LSCC‐related sequencing datasets for bioinformatics analysis. WGCNA was performed on GSE142083 and differential analysis was conducted on GSE51985 and TCGA‐HNSC. Intersection genes were taken from the above three datasets. To confirm the function of genes, we overexpressed and knocked down genes in cells and treated them with autophagy agonist Rapamycin and PI3K‐AKT pathway inhibitor. At the cellular level, the expression of CH25H, autophagy‐related proteins (LC3 I, LC3 II, p62, and Beclin 1), and PI3K‐AKT pathway‐related proteins (PI3K, AKT, and p‐AKT) were assessed via Western blot; the mRNA level of CH25H was evaluated through qRT‐PCR; the cell activity was examined by CCK8; the apoptosis was assessed through flow cytometry; and the cell migration and invasion were assessed through wound healing and Transwell assays.

**Results:**

Through bioinformatics analysis, we screened 7 genes (CH25H, NELL2, STC2, TMEM158, ZIC2, HOXD11, and HOXD10). Ultimately, CH25H was selected for follow‐up experiments. By detecting CH25H expression in human immortalized keratinocytes (HaCaT) and LSCC cells (Tu‐686, SNU899, and AMC‐HN‐8), it was found out that CH25H expression was higher in HaCaT cells than in LSCC cells. To elucidate the role of CH25H in LSCC development, we overexpressed CH25H in Tu‐686 cells and downregulated its expression in AMC‐HN‐8 cells. CH25H was revealed to reduce the proliferation, activity, invasion, and migration of LSCC cells while increasing their apoptosis levels. Significant changes were also observed in the expressions of autophagy‐ and PI3K‐AKT pathway‐related proteins. To further investigate the roles of autophagy and the PI3K‐AKT pathway in LSCC development, we respectively employed autophagy agonists and inhibitors targeting the PI3K‐AKT pathway to intervene the cells, and found that CH25H regulated the PI3K‐AKT pathway to promote autophagy, thus enhancing the apoptosis of LSCC cells. We further investigated CH25H's impact on tumor growth, autophagy, and the PI3K‐AKT pathway at the animal level and found that CH25H promoted autophagy of LSCC cells and inhibited the PI3K‐AKT pathway, and ultimately inhibiting the progression of LSCC.

**Conclusions:**

In summary, CH25H promotes autophagy and affects the malignant progression of LSCC through the PI3K‐AKT pathway.

**Abbreviations**
BCA: bicinchoninic acid
DEGs: differentially expressed genes
EMT: epithelial‐mesenchymal transition
GSEA: gene set enrichment analysis
HNSC: head and neck squamous cell carcinoma
LSCC: laryngeal squamous cell carcinoma
Rap: rapamycin
STR: short tandem repea

## 1 Introduction

Laryngeal cancer, as the malignant tumor within the neck and head following nasopharyngeal cancer and nasal cancer [1, 2], primarily manifests as laryngeal squamous cell carcinoma (LSCC), constituting approximately 90% of cases. LSCC is characterized by an insidious onset and highly variable early clinical symptoms and is susceptible to cervical lymph node metastasis. Notably, LSCC is one of the diseases with a reduced 5‐year survival rate [3]. Therefore, identifying new biomarkers assumes paramount significance for the diagnosis of LSCC.

WGCNA is a method used to identify genes with synergistic changes, enabling the screening of therapeutic targets or biomarker genes for any specific disease based on the correlation between phenotypes and gene sets. It is reported that WGCNA is specifically designed for screening the genetic modules of collaborative expression, investigating the core genes in the network, and analyzing the correlation between focal phenotype and gene network [4]. Additionally, WGCNA is a valuable approach for screening prognostic markers, disease markers, and drug therapeutic targets [5, 6]. Currently, WGCNA has been extensively applied in the studies of various diseases, including lung, liver, and pancreatic cancer [7, 8, 9].

In this study, we conducted WGCNA and differential analysis on LSCC‐related datasets (GSE142083 and GSE51985), and meanwhile performed differential analysis on the head and neck squamous cell carcinoma (HNSC) related datasets obtained from TCGA database. By identifying intersection genes from three datasets, we selected CH25H for functional analysis to screen out key pathways and investigate the molecular mechanisms associated with LSCC progression. Through a combination of experimental testing and bioinformatics analysis, we aim to provide a scientific basis for elucidating the potential mechanism of LSCC development and offer novel insights into the targeted drug therapy for LSCC.

## 2 Materials and Methods

### 2.1 Datasets

Using the “GEOquery” package, we retrieved two datasets, GSE142083 and GSE51985, from the GEO database. The GSE142083 dataset, comprised of FPKM data, encompasses 106 samples, including 53 from LSCC tissues and 53 from adjacent normal mucosa tissues. Gene IDs in the expression profile were converted into gene symbols in the Gencode database, resulting in annotations for a total of 19,328 genes. The GSE51985 dataset was preprocessed to screen out mRNA with expression values ≥ 0. A total of 20 samples were selected, including 10 from adjacent lung tumor tissues and 10 from LSCC tissues. Furthermore, we downloaded HNSC data from the TCGA database, with the data type set to “count.” According to the clinical information, 123 laryngeal cancer‐related samples were selected, including 111 laryngeal cancer samples and 12 normal samples.

### 2.2 WGCNA

WGCNA was performed using the “WGCNA” package (v 1.72.1).

### 2.3 Differential Analysis and Enrichment Analysis

Differential analysis was performed using the “limma” package (v 3.54.2). Genes meeting the criteria of adj.pVal < 0.05 and |log_2_FC| > 2 were considered as significant differentially expressed genes (DEGs). “Veen” was used for Wayne diagram construction for visualization.

### 2.4 Cell Culture and Treatment

We included human keratinocytes (HaCaT) in the control group and LSCC cells (AMC‐HN‐8, Tu‐686, and SNU899) in the experimental group for follow‐up experiments. HaCaT cells (Cell Bank of the Chinese Academy of Sciences, #SCSP‐5091) were cultured in high‐glucose DMEM (Gibco, #11995065) supplemented with 10% FBS. The human LSCCs TU‐686 (EK‐Bioscience, #CC‐Y1654), SNU899 (BioVector NTCC, #SNU‐899), and AMC‐HN‐8 (FENGHUISHENGWU, #CL0666) were cultured in RPMI‐1640 (Gibco, #11875119) with 10% FBS. All cells were identified by short tandem repeat (STR), and the cell lines were tested for mycoplasma contamination. All cells were cultured in constant temperature and humidity‐controlled incubators (37°C, 5% CO_2_). CH25H overexpression vectors were constructed and transfected into TU‐686 cells to establish the Tu‐686 + oe‐CH25H group, while empty plasmids were transfected into TU‐686 cells to establish the Tu‐686 + oe‐NC group (the control group). In addition, siRNA oligonucleotides targeting CH25H were transfected into AMC‐HN‐8 cells to construct the AMC‐HN‐8 + sh‐CH25H group, while negative siRNA oligonucleotides were transfected into AMC‐HN‐8 cells to construct the AMC‐HN‐8 + sh‐NC group (the control group).

### 2.5 Experimental Animal

Six‐week‐old healthy male BALB/c mice were purchased from the Hangzhou Medical College Laboratory Animal Center and were kept in a controlled environment (light/dark cycle: 12/12 h, humidity: 65%, temperature: 23°C). This animal experimental protocol has been reviewed by the Zhejiang Center of Laboratory Animals, in compliance with relevant provisions of the Institutional Animal Care and Use Committee of Zhejiang Center of Laboratory Animals (Approval number: ZJCLA‐IACUC‐20040153).

### 2.6 Tumor Formation and Grouping of Mice

A cell suspension with a concentration of 3 × 10^6^ cells/mL was prepared and subcutaneously injected into the right armpit of mice using a disposable sterile syringe. The tumor volume was measured at 7 d intervals postinjection. On the 28th day, the mice were euthanized, followed by dissection, removal, weighing, and photography of the tumors. The mouse tumor tissues injected with oe‐NC‐transfected TU‐686 cells were designated as the tumor+oe‐NC group, while those injected with oe‐CH25H‐transfected TU‐686 cells were labeled as the tumor+oe‐CH25H group.

### 2.7 qRT‐PCR

Total RNA was extracted using the TRIzol assay, followed by cDNA synthesis using the PrimeScript RT kit. Quantitative PCR was carried out with the SYBR Green PCR kit. The primer sequences are shown in Table 1.

**TABLE 1:** Primer sequences.

Primer	Sequence
CH25H‐forward (F)	5′‐GGCATACCAAGTGTCCTTCTAAGC‐3′
CH25H‐reverse (R)	5′‐AACACCCTACACCCAGATTCCTC‐3′
GAPDH‐F	5′‐TGAAGCAGGCATCTGAGGG‐3′
GAPDH‐R	5′‐CGAAGGTGGAA GAGTGGGAG‐3′

### 2.8 WB

The cell lysate containing 1% protease inhibitor was used for the extraction of total proteins from both cells and placental tissues. The quantification of the extracted protein samples was performed using the bicinchoninic acid (BCA) method, followed by electrophoresis, membrane transfer, antibody incubation, and coloration. The primary antibodies used included CH25H (ThermoFisher, # PA5‐72349), LC3 I (Affinity Biosciences, # AF4007), LC3 II (Affinity Biosciences, # AF4650), p62 (Abcam, #ab109012), Beclin 1 (Abcam, # ab207612), PI3K (cell signaling technology, CST, # 3811), AKT (CST, #9272), and p‐AKT (CST, #9271). GAPDH (Abcam, # ab9485) was selected as the internal reference gene.

### 2.9 CCK8

A total of 6000 cells per well were inoculated into 12‐well plates and cultured for 48 h. Subsequently, CCK8 reagent (Solarbio, #CA1210) was added and further incubation was continued at 37°C for 2 h. Then, the detection area was selected and the wavelength of the enzyme labeler was set at 450 nm to detect the value of optical density for the evaluation of cell activity.

### 2.10 Colony Formation

The cells were inoculated into 6‐well plates with 1000 cells/well, and cultured until the cell counts in most monoclones exceeding 50. The cells were washed, fixed with 4% paraformaldehyde, and stained with crystal violet dye before being photographed.

### 2.11 Detection of Apoptosis

A total of 5 × 10^5^ cells were collected and resuspended in 1 × Annexin V Binding Buffer. Next, 7‐AAD staining solution and Annexin V‐APC were added, and the cells were incubated in the dark. Apoptosis was assessed using the apoptosis kit purchased from Pricella (product number: P‐CA‐201).

### 2.12 Scratch Wound Healing

The cells were cultured in vitro to form a monolayer adherent. Cells in the central area were subsequently removed, and the remaining cells were further cultured with serum‐free medium. Then, the cell culture plate was removed, and the migration of the surrounding cells to the central scratched area was assessed by observation under a microscope.

### 2.13 Transwell

The Matrigel matrix glue (Solarbio, #G4740) was applied to the bottom of the upper Transwell chamber. After solidification, cell suspension was added to the lower compartment, and the medium was added to the lower chamber. After routine culture in the cell incubator for 24 h.

### 2.14 Statistical Analysis

The statistical analysis of the bioinformatics data was carried out using R. For experimental data, we performed statistical analysis and generated bar graphs using GraphPad prism 9.0. The measurement data were expressed as mean ± standard deviation ($\bar{x}\pm \mathrm{SD}$). The *t*‐test was used to assess the differences between groups.

## 3 Results

### 3.1 WGCNA

**FIGURE 1:**
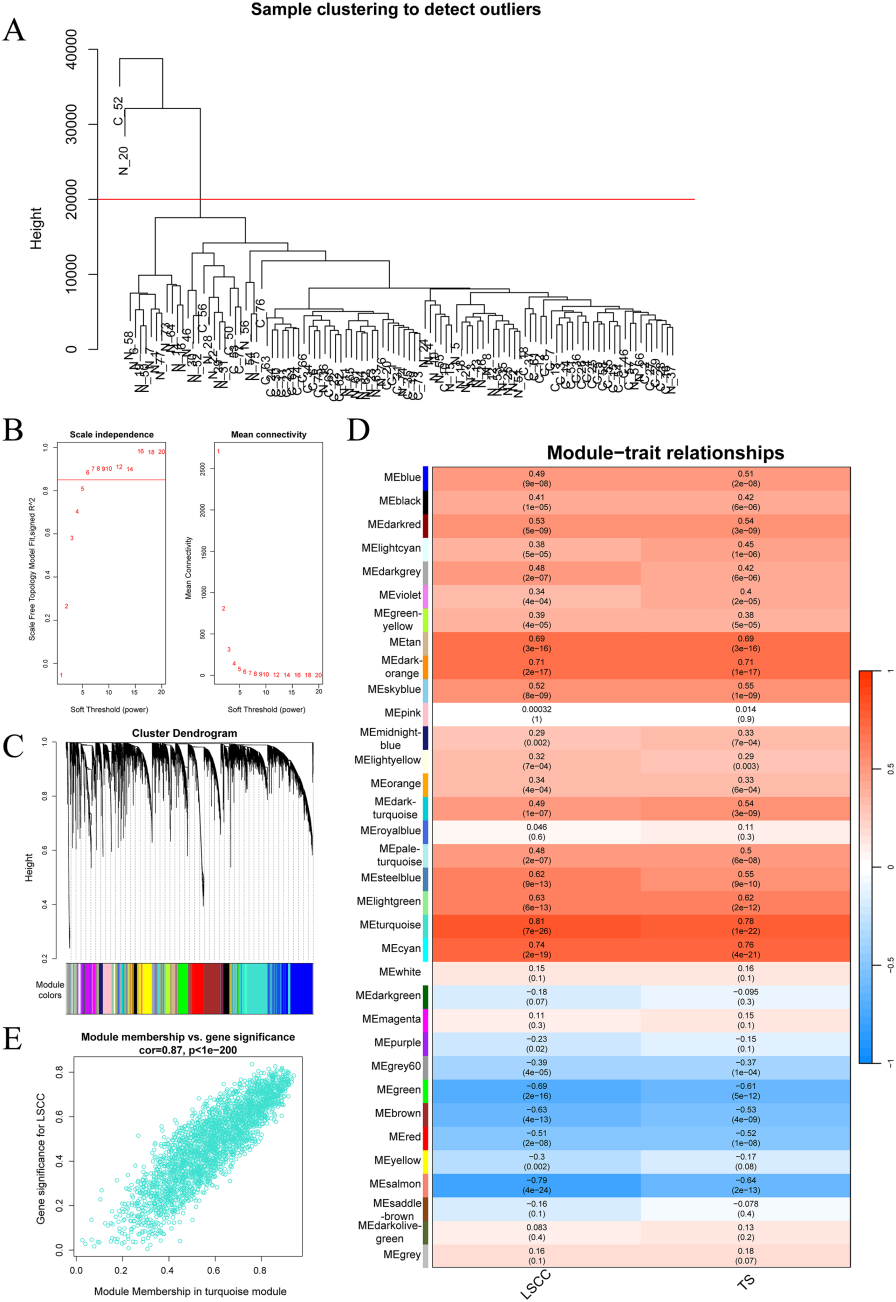
WGCNA (A) Cluster phylogenetic tree of the samples; (B) Analysis of the scale‐free fit index and the mean connectivity for various soft‐thresholding powers; (C) Correlation between clinical status and module Eigengenes; (D) Correlation heatmap of modules and features; (E) Correlation heatmap of the turquoise module and LSCC.

We preprocessed the GSE142083 dataset, selecting the top 75% genes based on the median absolute deviation (14,496 genes). Subsequently, we conducted sample clustering and removed two outlier samples (Figure 1A). Next, we performed a topological analysis on the data (Figure 1B), clustered the genes with a soft threshold of 8 (Figure 1C). According to the correlation analysis between groups and modules (Figure 1D), the turquoise module with *p* < 0.05 and the highest correlation was selected. The scatter plot shows a significant positive correlation between the LSCC and the turquoise module (Figure 1E). Therefore, the turquoise module, which includes 2337 genes, was selected for subsequent analysis.

### 3.2 Screening for Key DEGs

**FIGURE 2:**
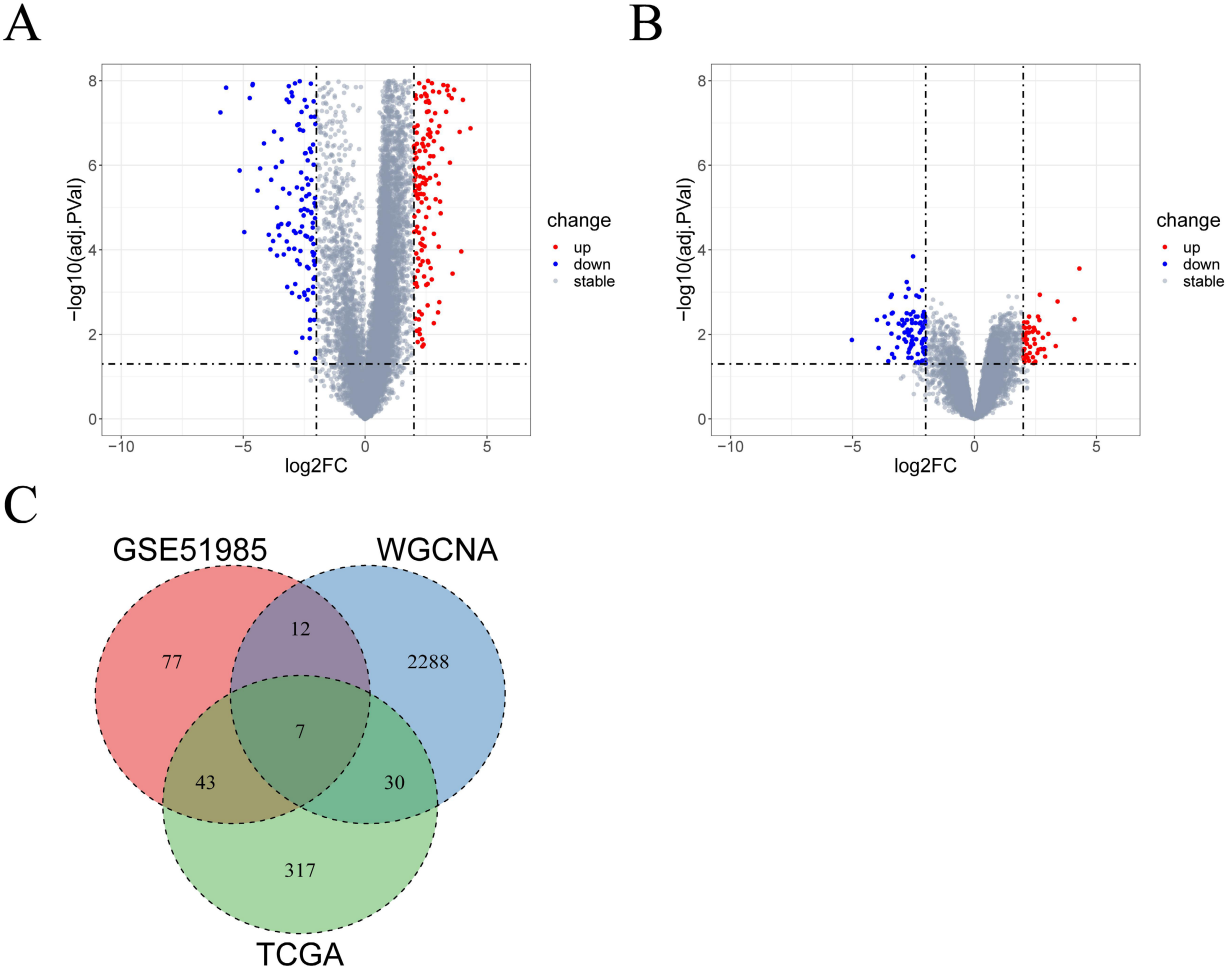
Screening for key DEGs (A) The volcano plot showing the DEGs from the TCGA‐HNSC dataset; (B) The volcano plot showing the DEGs from the GSE51985 dataset; (C) The Venn diagram showing the key DEGs screened from TCGA‐HNSC, GSE51985, and WGCNA.

We performed differential analyses respectively on HNSC‐related data (TCGA‐HNSC) and on the GSE51985 dataset, with both comparative strategy as cancer samples vs. normal samples. Based on |log_2_FC| > 2 and adj.PVal < 0.05, 397 DEGs (Figure 2A) and 139 DEGs (Figure 2B) were screened out accordingly. We made an intersection of genes from the turquoise module, DEGs from the TCGA‐HNSC dataset, and DEGs from the GSE51985 dataset, ultimately identifying seven key DEGs: CH25H, NELL2, STC2, TMEM158, ZIC2, HOXD11, and HOXD10 (Figure 2C).

### 3.3 Functional Enrichment Analysis

**FIGURE 3:**
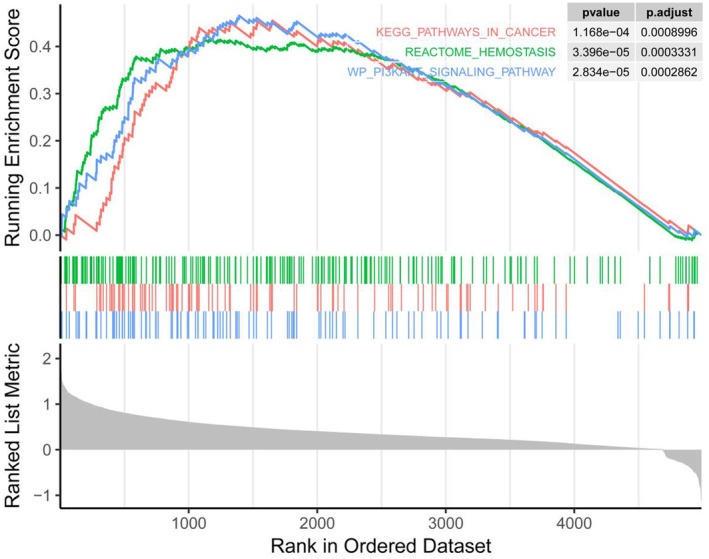
WGCNA.

Based on relevant literature review, CH25H has been confirmed to be related to tumor immune escape and tumor microenvironment. However, CH25H remains currently relatively poorly studied in tumors, leaving uncertainty regarding whether CH25H may regulate the malignant progression of tumors through other pathways. Therefore, we chose CH25H for follow‐up experiments. Through gene set enrichment analysis (GSEA), we found that CH25H affected the hemostasis reaction and the PI3K/AKT pathway (Figure 3).

### 3.4 CH25H Expression in LSCC Cells

**FIGURE 4:**
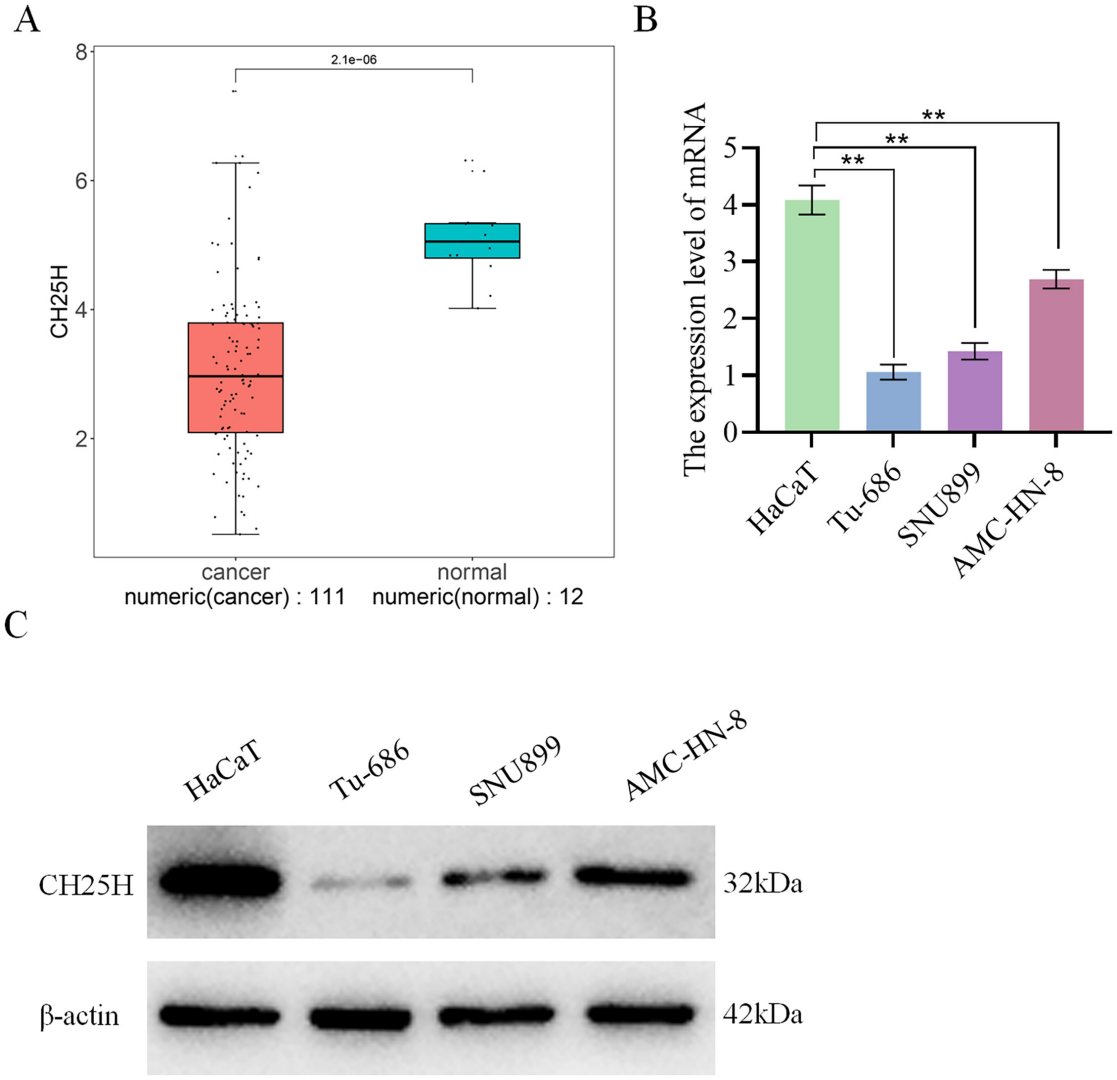
CH25H expression in LSCC cells (A) CH25H expression in the TCGA‐HNSC dataset; (B) The relative expression level of CH25H in LSCC cells; (C) The expression of CH25H (**p < 0.01).

Analysis of CH25H expression in the TCGA‐HNSC dataset revealed significant lower expression of CH25H in cancer samples compared with normal samples (Figure 4A). qRT‐PCR analysis demonstrated a substantial decrease in CH25H expression in AMC‐HN‐8, SNU899, and Tu‐686 compared with HaCaT, with a sequential reduction observed in AMC‐HN‐8, SNU899, and Tu‐686 (Figure 4B). WB experiments further revealed a significant reduction in CH25H expression in AMC‐HN‐8, SNU899, and Tu‐686 relative to HaCaT (Figure 4C). These results indicate that CH25H was lowly expressed in LSCC cells.

### 3.5 CH25H Inhibits the Proliferation of LSCC Cells

**FIGURE 5:**
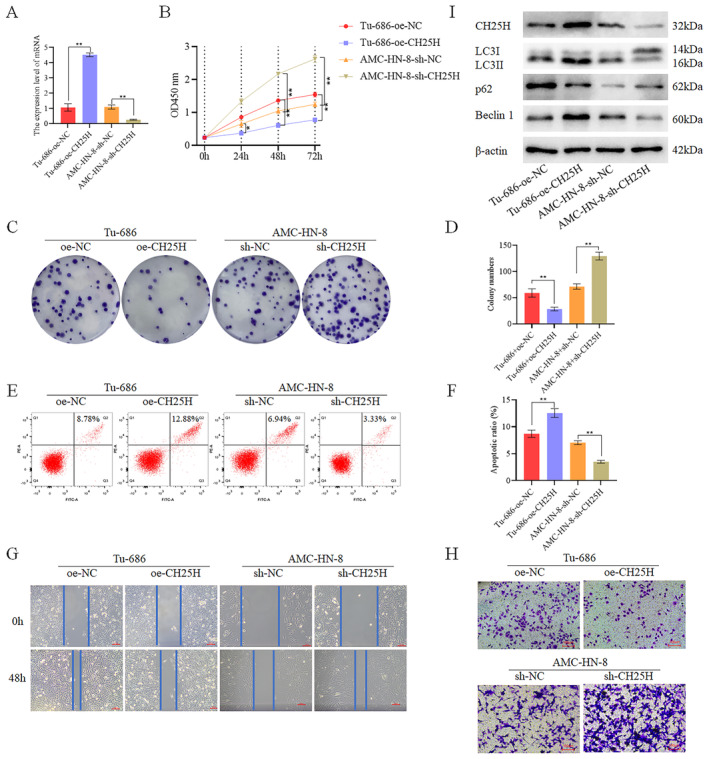
CH25H inhibited the proliferation of LSCC cells (A) After overexpressing CH25H in TU‐686 and knocking down CH25H in AMC‐HN‐8, the expression of CH25H was detected; (B) After TU‐686 were overexpressed with CH25H and AMC‐HN‐8 were knocked down with CH25H, the cell viability was detected; (C) After TU‐686 were overexpressed with CH25H and AMC‐HN‐8 were knocked down with CH25H, the cell colony formation was detected; (D) The cell proliferation of each group was displayed by the bar chart; (E) After TU‐686 were overexpressed with CH25H and AMC‐HN‐8 were knocked down with CH25H, the cell apoptosis was detected; (F) The apoptosis level of each group was displayed by the bar chart; (G) After TU‐686 were overexpressed with CH25H and AMC‐HN‐8 were knocked down with CH25H, the migration was detected; (H) After TU‐686 were overexpressed with CH25H and AMC‐HN‐8 were knocked down with CH25H, the invasion was detected; (I) After overexpressing CH25H in TU‐686 and knocking down CH25H in AMC‐HN‐8, the expression of autophagy‐related proteins was detected by WB (**p < 0.01).

To investigate the role of CH25H, we overexpressed CH25H in Tu‐686 cells (designated as the TU‐686 + oe‐CH25H group) and knocked down CH25H in AMC‐HN‐8 (designated as the AMC‐HN‐8 + sh‐CH25H group) (Figure 5A,I). Assay revealed a decrease in cell activity in the TU‐686 + oe‐CH25H compared with the TU‐686 + oe‐NC. Conversely, cell activity was significantly increased in the AMC‐HN‐8 + sh‐CH25H relative to the AMC‐HN‐8 + sh‐NC (Figure 5B). Colony formation assay demonstrated a notable decrease in cell proliferation in the Tu‐686 + oe‐CH25H compared with the Tu‐686 + oe‐NC, while the AMC‐HN‐8 + sh‐CH25H exhibited increased cell proliferation in comparison with the AMC‐HN‐8 + sh‐NC (Figure 5C,D). Additionally, apoptosis experiments revealed substantially higher apoptosis levels in the Tu‐686 + oe‐CH25H compared with the Tu‐686 + oe‐NC, whereas the apoptosis level was significantly lower in the AMC‐HN‐8 + sh‐CH25H compared with the AMC‐HN‐8 + sh‐NC (Figure 5E,F). Scratch wound healing assay (Figure 5G) and Transwell assay (Figure 5H) demonstrated significantly lower levels of migration and invasion in the Tu‐686 + oe‐CH25H as compared to the Tu‐686 + oe‐NC, while higher levels of migration and invasion were revealed in the AMC‐HN‐8 + sh‐CH25H in comparison with the AMC‐HN‐8 + sh‐NC. Therefore, it was concluded that CH25H inhibits the migration, proliferation, invasion, and activity, migration of LSCC cells, while also promoting the apoptosis of these cells. Concurrently, WB analysis revealed the expression of autophagy‐related proteins. Specifically, the expression of Beclin 1 and LC3II/LC3I was higher in the Tu‐686 + oe‐CH25H than in the Tu‐686 + oe‐NC, but lower in the AMC‐HN‐8 + sh‐CH25H than in the AMC‐HN‐8 + sh‐NC. Moreover, the expression of p62 was lower in the Tu‐686 + oe‐CH25H than in the Tu‐686 + oe‐NC, but higher in the AMC‐HN‐8 + sh‐CH25H than in the AMC‐HN‐8 + sh‐NC (Figure 5I and Figures S1 and S4). These results suggest that CH25H may promote autophagy, leading to the inhibition of LSCC cell activity, proliferation, invasion, and migration, while simultaneously enhancing LSCC cell apoptosis, thereby affecting LSCC progression.

### 3.6 CH25H Affects Autophagy

**FIGURE 6:**
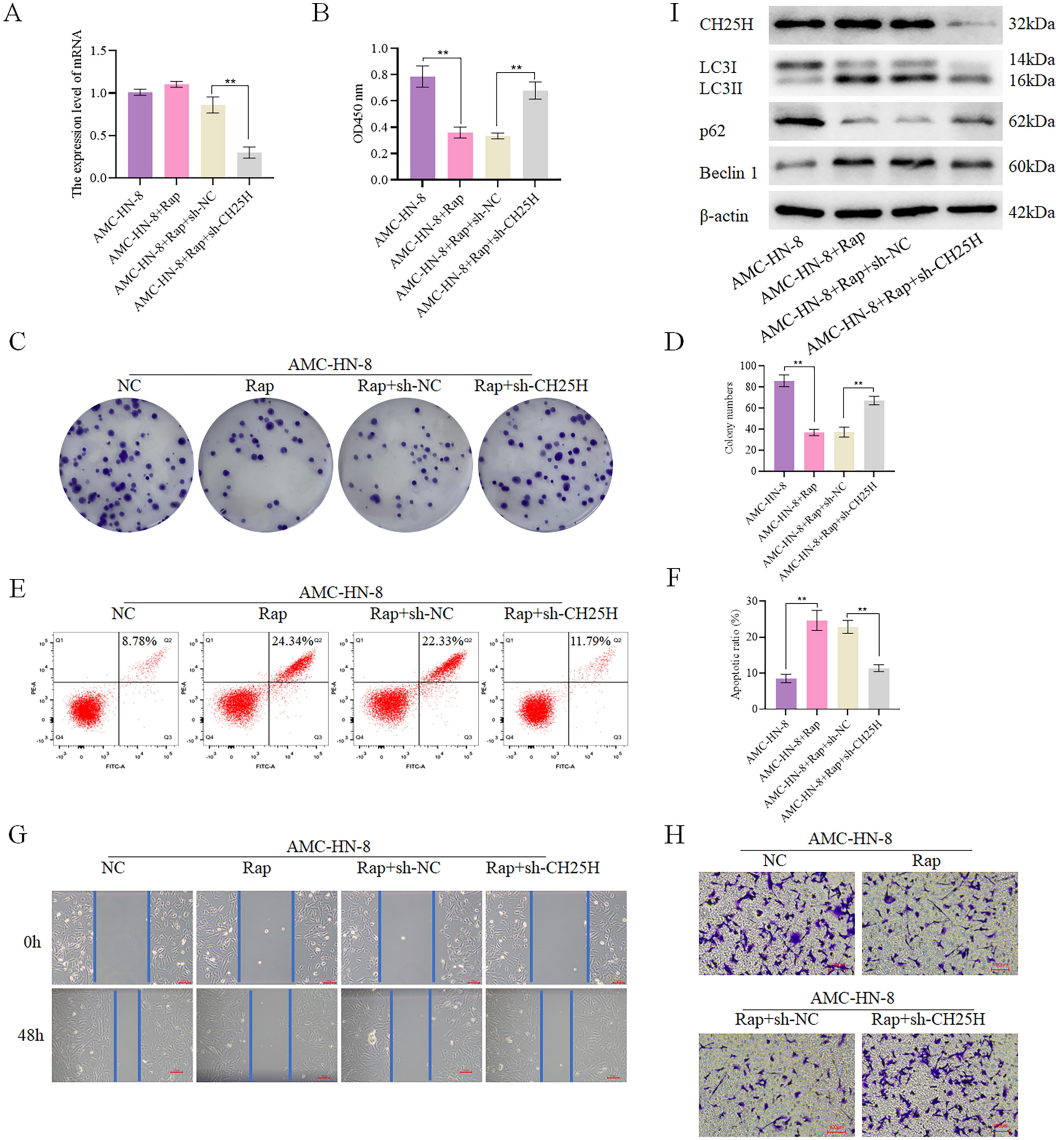
CH25H affected autophagy (A) After treating cells with autophagy agonists, the expression of CH25H was detected; (B) After treating cells with autophagy agonists, the cell viability was detected; (C) After treating cells with autophagy agonists, the cell colony formation was detected; (D) The proliferation in each group was displayed by the bar chart; (E) After treating cells with autophagy agonists, the cell apoptosis was detected; (F) The apoptosis level was displayed by the bar chart; (G) After treating cells with autophagy agonists, the cell migration was detected; (H) After treating cells with autophagy agonists, the cell invasion was detected; (I) After treating cells with autophagy agonists, the expression of autophagy‐related proteins was detected by WB (**p < 0.01).

To validate the impact of CH25H on autophagy, autophagy agonist rapamycin (Rap) was used to treat AMC‐HN‐8 cells with CH25H knockdown (Figure 6A&I). We set up the following groups: AMC‐HN‐8, AMC‐HN‐8 + Rap, AMC‐HN‐8 + Rap+sh‐NC, and AMC‐HN‐8 + Rap+sh‐CH25H. CCK8 assay revealed significantly lower cell viability in the AMC‐HN‐8 + Rap compared with the AMC‐HN‐8, and significantly higher cell viability in the AMC‐HN‐8 + Rap+sh‐CH25H compared with the AMC‐HN‐8 + Rap+sh‐NC (Figure 6B). Additionally, Assay indicated significantly lower levels of cell proliferation in the AMC‐HN‐8 + Rap relative to the AMC‐HN‐8, and significantly higher levels of cell proliferation in the AMC‐HN‐8 + Rap+sh‐CH25H relative to the AMC‐HN‐8 + Rap+sh‐NC (Figure 6C,D). Apoptosis assay showed a higher apoptosis level in the AMC‐HN‐8 + Rap by contrast with the AMC‐HN‐8, and lower apoptosis level in the AMC‐HN‐8 + Rap+sh‐CH25H when compared to the AMC‐HN‐8 + Rap+sh‐NC (Figure 6E,F). Furthermore, scratch wound healing (Figure 6G) and Transwell (Figure 6H) demonstrated lower levels of cell invasion and migration in the AMC‐HN‐8 + Rap compared with the AMC‐HN‐8, and significantly enhanced cell invasion and migration in the AMC‐HN‐8 + Rap+sh‐CH25H relative to the AMC‐HN‐8 + Rap+sh‐NC. Meanwhile, we detected the expression of autophagy‐related proteins, revealing that the expression of Beclin 1 and LC3II/LC3I was higher in the AMC‐HN‐8 + Rap than in the AMC‐HN‐8, and lower in the AMC‐HN‐8 + Rap+sh‐CH25H than in the AMC‐HN‐8 + Rap+sh‐NC. Additionally, the expression of p62 was lower in the AMC‐HN‐8 + Rap than in the AMC‐HN‐8, and higher in the AMC‐HN‐8 + Rap+sh‐CH25H than in the AMC‐HN‐8 + Rap+sh‐NC (Figure 6I, Figures S2, and S5). In conclusion, autophagy reduces the migration, viability, invasion, and proliferation of LSCC cells, and enhances their apoptosis, while downregulation of CH25H expression compensates for the effects of Rap. Therefore, CH25H can inhibit the activity, proliferation, invasion, and migration and promote the apoptosis of LSCC cells, by promoting autophagy, thereby affecting the development of LSCC.

### 3.7 CH25H Promotes the Autophagy of LSCC Cells Through the PI3K‐AKT Pathway

**FIGURE 7:**
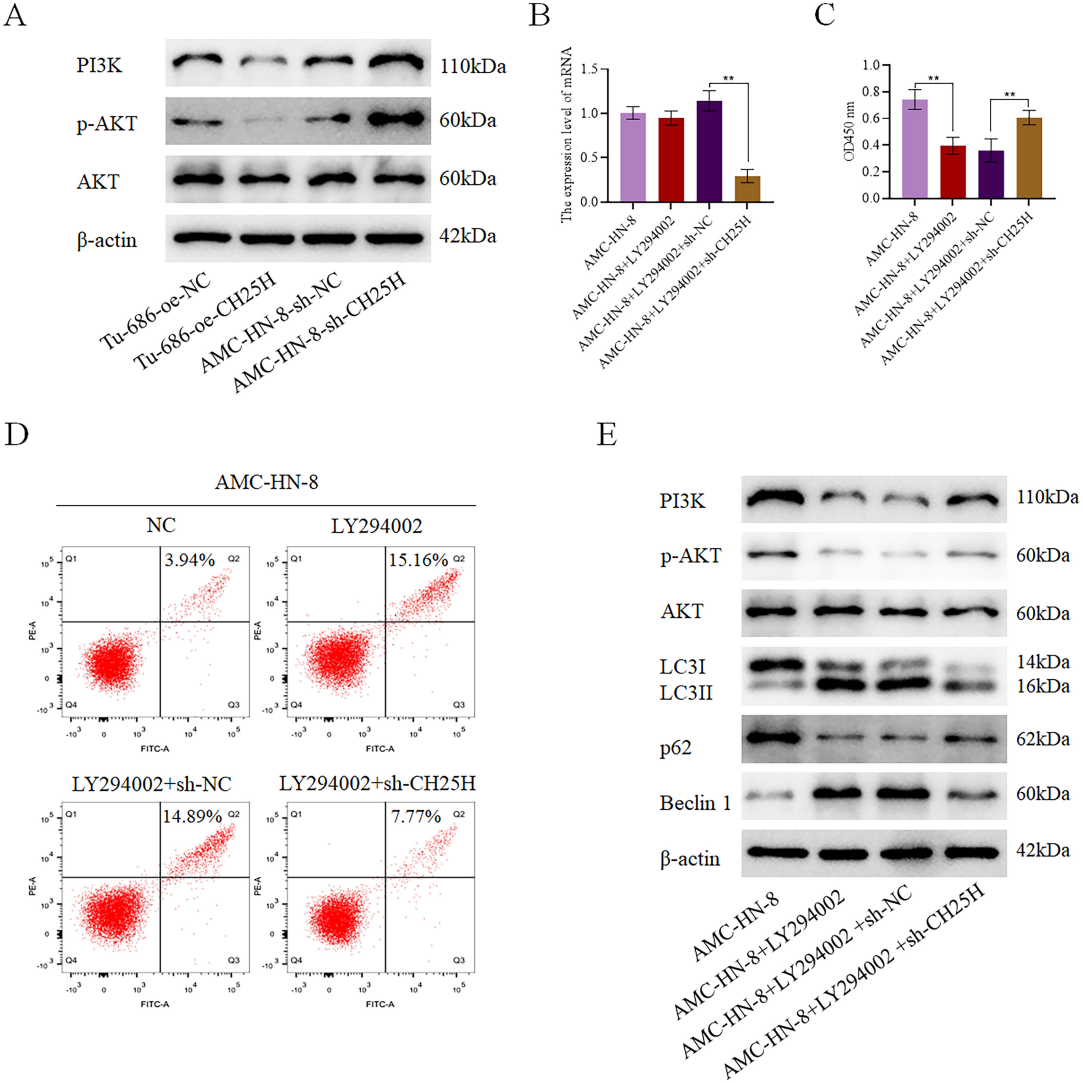
CH25H promoted the autophagy of LSCC cells through the PI3K‐AKT pathway (A) The expressions of PI3K‐AKT pathway‐related proteins in cells; (B) The relative expression of CH25H in each group of cells; (C) The cell viability; (D) The apoptosis; (E) The expressions of PI3K‐AKT pathway and autophagy‐related proteins in cells (**p < 0.01).

Based on our previous bioinformatics analysis, CH25H might regulate the PI3K‐AKT pathway. For further verification, we detected the expression of key proteins related to the PI3K‐AKT pathway in Tu‐686 with CH25H overexpression and AMC‐HN‐8 with CH25H downregulation. The expression levels of p‐AKT and PI3K were revealed to be lower in the Tu‐686 + oe‐CH25H than in the Tu‐686 + oe‐NC, and higher in the AMC‐HN‐8 + sh‐CH25H than in the AMC‐HN‐8 + sh‐NC (Figure 7A). In addition, LY294002, an inhibitor of the PI3K‐AKT pathway, was added to the AMC‐HN‐8 + sh‐CH25H, after which the expression of CH25H was detected in each group (Figure 7B). We set up the following groups: AMC‐HN‐8, AMC‐HN‐8 + LY294002, AMC‐HN‐8 + LY294002 + sh‐NC, and AMC‐HN‐8 + LY294002 + sh‐CH25H. By performing CCK8 assay, we observed lower levels of cell viability in the AMC‐HN‐8 + LY294002 compared with the AMC‐HN‐8 and higher levels of cell viability in the AMC‐HN‐8 + LY294002 + sh‐CH25H compared with the AMC‐HN‐8 + LY294002 + sh‐NC (Figure 7C). Apoptosis assay further demonstrated significantly higher levels of apoptosis in the AMC‐HN‐8 + LY294002 relative to the AMC‐HN‐8 and significantly lower levels of apoptosis in the AMC‐HN‐8 + LY294002 + sh‐CH25H relative to the AMC‐HN‐8 + LY294002 + sh‐NC (Figure 7D). Additionally, WB results showed higher expression levels of Beclin 1 and LC3II/LC3I in the AMC‐HN‐8 + LY294002 compared with the AMC‐HN‐8, a noteworthy reduction in the AMC‐HN‐8 + LY294002 + sh‐CH25H compared with the AMC‐HN‐8 + LY294002 + sh‐NC. Meanwhile, the expression levels of p62, p‐AKT, and PI3K were decreased in the AMC‐HN‐8 + LY294002 in comparison with the AMC‐HN‐8, but were notably increased in the AMC‐HN‐8 + LY294002 + sh‐CH25H when compared to the AMC‐HN‐8 + LY294002 + sh‐NC (Figure 7E, Figures S3, and S6). In summary, these findings suggest that CH25H promotes autophagy of LSCC cells by inhibiting the PI3K‐AKT pathway to inhibit the occurrence and development of LSCC.

### 3.8 CH25H Inhibits LSCC Progression

**FIGURE 8:**
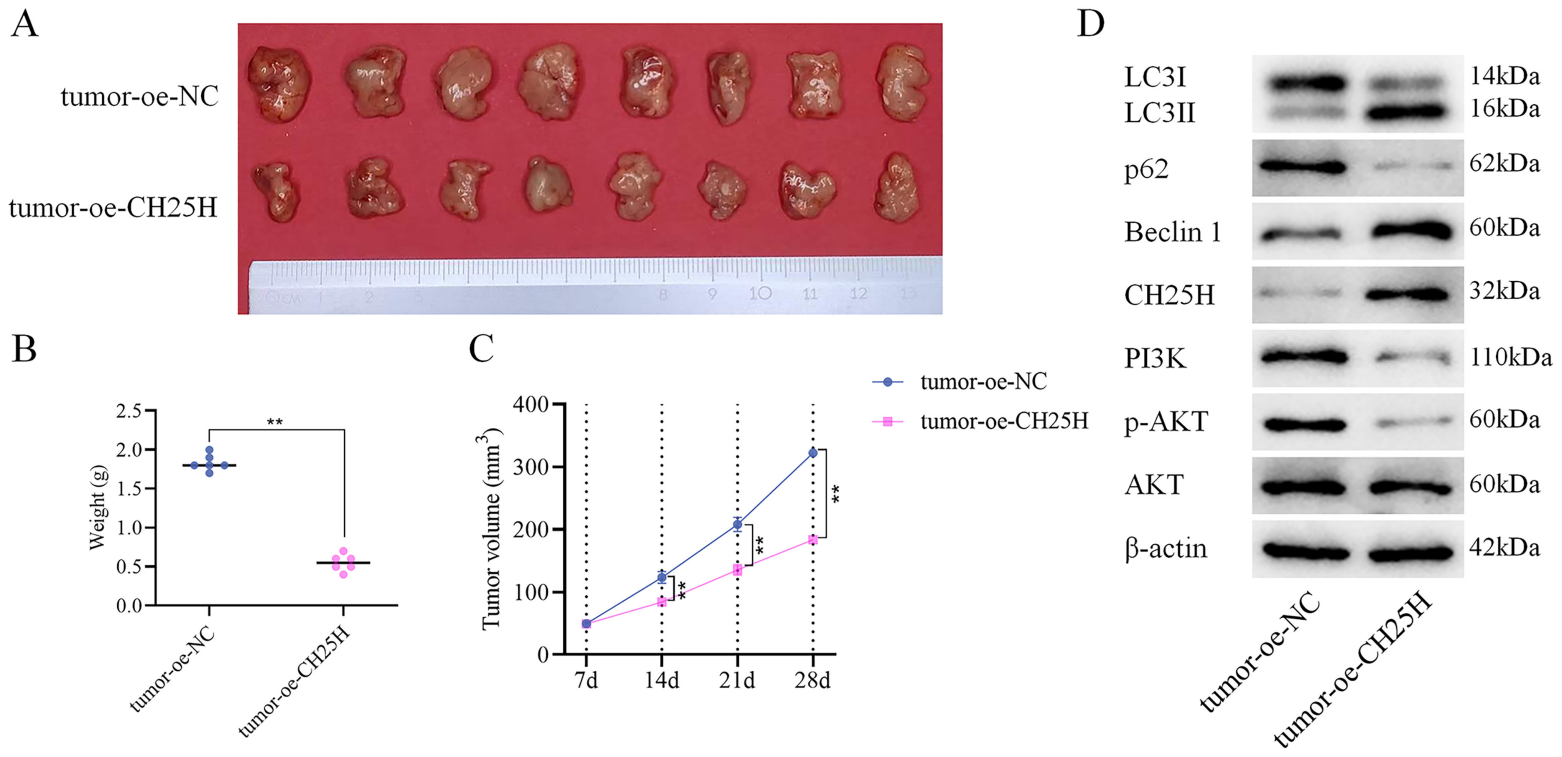
CH25H inhibited the tumor growth of LSCC (A) The tumor size in each group (*n* = 6); (B) The average tumor weight of different groups (*n* = 6); (C) The average tumor size of different groups (*n* = 6); (D) The expressions of CH25H, PI3K‐AKT pathway, and autophagy‐related proteins in cells (**p < 0.01).

In our nude mouse transplantation tumor experiment, we assessed the impact of CH25H on tumor growth. The experimental results demonstrated the inhibitory effects of CH25H on tumor growth (Figure 8A), as evidenced by reductions in both tumor weight (Figure 8B) and volume (Figure 8C). Furthermore, WB analysis revealed higher expression levels of CH25H, Beclin 1, LC3II/LC3I proteins, and lower expression levels of p‐AKT, PI3K, p62 proteins in the tumor+oe‐CH25H compared with the tumor+oe‐NC (Figure 8D). In conclusion, CH25H was demonstrated to effectively suppress LSCC progression via inhibiting the PI3K‐AKT pathway and promoting the autophagy of LSCC cells.

## 4 Discussion

LSCC typically presents with primary symptoms such as hoarseness, dyspnea, cough, and dysphagia. Our goal was to find effective strategies to delay the progression of LSCC and facilitate its early detection.

By performing WGCNA on the downloaded LSCC‐related dataset, GSE142083, the turquoise module, comprising 2337 genes, was identified with a strong correlation with LSCC. Thorough subsequent differential analyses on the TCGA‐HNSC data and the GSE51985 dataset, 397 and 139 DEGs were screened out, respectively. After interesting the genes obtained from the three datasets, seven key DEGs (CH25H, NELL2, STC2, TMEM158, ZIC2, HOXD11, and HOXD10) were identified. CH25H is an oxysterol that regulates lipid metabolism and plays a variety of functions in immune responses, regulating cholesterol homeostasis, and inflammation [10]. It activates the NLRP3 inflammasome and promotes the occurrence of liver steatosis [11, 12]. In the context of cancer, CH25H serves as a risk stratification biomarker for lung adenocarcinoma [13] and pancreatic cancer [14]. Depletion of CH25H has been shown to promote an increase in neutrophils in tumor‐specific cytotoxic T lymphocytes, weaken antitumor immunity, and stimulate tumor growth [15]. CH25H can also inhibit extracellular vesicle intake and extracellular vesicle‐mediated tumor growth, premetastatic microenvironment formation, and lung cancer metastasis [16]. Furthermore, experiments involving mouse dendritic cells have highlighted the impact of CH25H on tumor immune evasion [17]. NELL2 is expressed in nervous tissues, and plays a role in regulating the differentiation of neurons, polarization, and axon guidance [18]. It has also been identified as a suppressor of gastric cancer [19] and breast cancer [20] development. STC2, a secretory glycoprotein, plays a role in regulating calcium, glucose homeostasis, and phosphorus transfer [21]. It is expressed in a variety of tumor cells and tumor tissues, including liver cancer, esophageal cancer, lung cancer, kidney cancer, gastric cancer, bone cancer, and breast cancer [22]. In addition, STC2 can promote the proliferation of pancreatic cancer cells by promoting epithelial‐mesenchymal transition (EMT) of pancreatic cancer cells [23]. TMEM158 has also been reported to affect tumor growth [24]. Studies have revealed that TMEM158 can activate the TGF‐β1 and PI3K/AKT pathways, thereby promoting the invasion of pancreatic cancer [25]. Additionally, TMEM158 has been demonstrated to be associated with the enhancement of proliferation of glioblastoma cells through the STAT3 signaling pathway [26]. ZIC2 has shown its involvement in the development of colorectal [27], breast [28], nonsmall cell lung [29], and prostate cancer [30]. HOXD11 and HOXD10, being homologous proteins, play distinct roles in cancer biology. HOXD11 has shown promise as a prognostic biomarker capable of predicting advanced diseases with a poor prognosis, and HOXD11 is also able to stimulate penile squamous cell carcinoma [31] and esophageal squamous cell carcinoma [32], while HOXD10 is a tumor suppressor in pancreatic cancer, inhibiting tumor cell invasion and EMT [33].

We conducted a literature review related to these seven genes during the past 5 years, and finally chose CH25H as the research object for subsequent experimental verification. As revealed by GSEA, CH25H could affect hemostasis and the PI3K/AKT signaling pathway, which has not been reported by relevant literature. Tumor patients have a high incidence of thrombotic complications, and present with main symptoms of hemostasis system disorders and coagulation abnormalities. It has been confirmed that the hemostatic system can regulate multiple immune functions and jointly drive tumorigenesis [34]. Thrombosis is closely related to the hemostasis mechanism, and it affects tumor development, angiogenesis, and metastatic spread [35]. Similarly, the presence of cancer cells also increases the risk of thrombosis [36]. By activating platelets, cancer cells regulate hematopoietic function and drive the migration of immune cells to the tumor site, thereby promoting the occurrence of cancer‐related inflammation [37]. According to existing literature, changes in the levels of hemostatic biomarkers in peripheral blood reflect systemic coagulation levels in cancer patients, serving as biomarkers indicative of underlying cancer activity and progression dynamics [38]. Consequently, hemostasis has a broad research prospect in the clinical evaluation of tumor stage. The PI3K/Akt pathway not only regulates cell autophagy, growth, invasion, and angiogenesis [39], but also the development of a variety of cancers [40], such as pancreatic cancer [41], esophageal cancer [42], and laryngeal squamous cell carcinoma [43, 44]. As reported, extracts from marine microorganisms, plants and animals or their metabolites can exert antitumor effects by regulating the PI3K/Akt pathway [45]. Therefore, investigation of the PI3K/Akt pathway is of great importance for the clinical treatment of cancer patients, including those with LSCC.

Bioinformatics analysis showed significantly lower levels of CH25H expression in the LSCC samples within the TCGA‐HNSC dataset. Moreover, the relative expression level of CH25H in LSCC cells was lower than that in HaCaT cells in vitro. To investigate the regulatory mechanism of CH25H, we conducted experiments involving the knockdown and overexpression of CH25H in LSCC cells. We hypothesized that CH25H could suppress the malignant progression of LSCC cells by inhibiting the PI3K‐AKT pathway and promoting autophagy of LSCC cells. Autophagy acts as a tumor promoter or suppressor depending on the different stages of tumors [46]. It is worth noting that CH25H has been revealed to inhibit avian leukosis virus subgroup J infection by regulating autophagy in the chick embryo fibroblast cell line [12]. In LSCC‐related studies, circPARD3 has been identified as an inhibitor of autophagy, driving the malignant progression of LSCC, and contributing to chemoresistance [47]. Furthermore, Niraparib, an antitumor drug PARP inhibitor used in clinical practice, also targets LSCC by affecting autophagy [48]. Moreover, by regulating the PI3K/AKT pathway, YBX1 promotes the malignant progression of LSCC cells [44], while Tra2 beta advances LSCC proliferation [49]. Similarly, in other types of cancers, Tanshinone I induces autophagy by inhibiting the PI3K/AKT/mTOR pathway, thereby mitigating the malignant biological characteristics of ovarian cancer [50]. Reportedly, via regulating the PI3K/AKT pathway, PHLDA2 affects EMT and autophagy, and inhibits the growth of colon cancer [51], and SOCS5 regulates autophagy pathway and promotes the transfer of liver cancer cells [52]. Finally, we conducted animal‐level experiments to validate the function by which CH25H inhibits the PI3K‐AKT pathway and induces autophagy in LSCC cells to inhibit LSCC progression.

In this study, by performing bioinformatics analysis on the datasets downloaded from publicly available databases: GEO and TCGA, CH25H was identified as a possible key factor in LSCC development. In addition, it was revealed at both cellular and animal levels that CH25H can suppress LSCC progression by inhibiting the PI3K‐AKT pathway and promoting the occurrence of autophagy. This study facilitates further understanding of the pathological mechanism of LSCC, providing biomarkers for the diagnosis of LSCC, and also a scientific theoretical basis for the development of drugs for treating LSCC. Clinically, the situation of patients with LSCC is more complicated. According to previous studies, the occurrence of LSCC is related to uncontrollable factors such as lifestyle, gender, and age, and there are individual differences. Therefore, it is unclear whether it can play a significant role in the clinical treatment and delay of the development of LSCC patients. We still need to collect more clinical samples to confirm our findings.

## Author Contributions

**Zhenfei Xiang:** conceptualization (lead), funding acquisition (lead), project administration (lead), resources (lead), writing – original draft (lead). **Senquan Yu:** formal analysis (equal), methodology (lead), software (equal), writing – original draft (supporting). **Yuxin Xu:** data curation (equal), formal analysis (equal), investigation (equal), visualization (equal). **Huacai Xiong:** data curation (equal), formal analysis (equal), investigation (equal), validation (equal), visualization (equal). **Danfei Hu:** data curation (equal), formal analysis (equal), investigation (equal), methodology (equal), software (equal), visualization (equal). **Qun Li:** formal analysis (equal), investigation (equal), software (equal), supervision (equal), validation (equal), visualization (equal). **Zhenhua Wu:** conceptualization (lead), funding acquisition (lead), project administration (lead), resources (lead), supervision (lead), writing – review and editing (equal).

## Ethics Statement

The animal experimental protocol has been reviewed by the Zhejiang Center of Laboratory Animals, in compliance with relevant provisions of the Institutional Animal Care and Use Committee of Zhejiang Center of Laboratory Animals (Approval number: ZJCLA‐IACUC‐20040153).

## Conflicts of Interest

The authors declare no conflicts of interest.

## Supporting information


**Figure S1.** After overexpressing CH25H in TU‐686 and knocking down CH25H in AMC‐HN‐8, the localization of autophagy‐related proteins was detected by immunofluorescence.

**Figure S2.** After treating cells with autophagy agonists, the localization of autophagy‐related proteins was detected by immunofluorescence.

**Figure S3.** After treating cells with LY294002, the localization of autophagy‐related proteins was detected by immunofluorescence.

**Figure S4.** After overexpressing CH25H in TU‐686 and knocking down CH25H in AMC‐HN‐8, the localization of autophagy‐related proteins was detected by electron microscope.

**Figure S5.** After treating cells with autophagy agonists, the localization of autophagy‐related proteins was detected by electron microscope.

**Figure S6.** After treating cells with LY294002, the localization of autophagy‐related proteins was detected by electron microscope.

## Data Availability

The data generated during the current study are included in this article, and further information can be available from the corresponding author upon reasonable request.
