# Supplementary figures and images for: CH25H Promotes Autophagy and Regulates the Malignant Progression of Laryngeal Squamous Cell Carcinoma Through the PI3K‐AKT Pathway
Source: Cancer Med. 2024 Oct 21;13(20):e70312. doi: 10.1002/cam4.70312 (PMC11491687; doi:10.1002/cam4.70312)

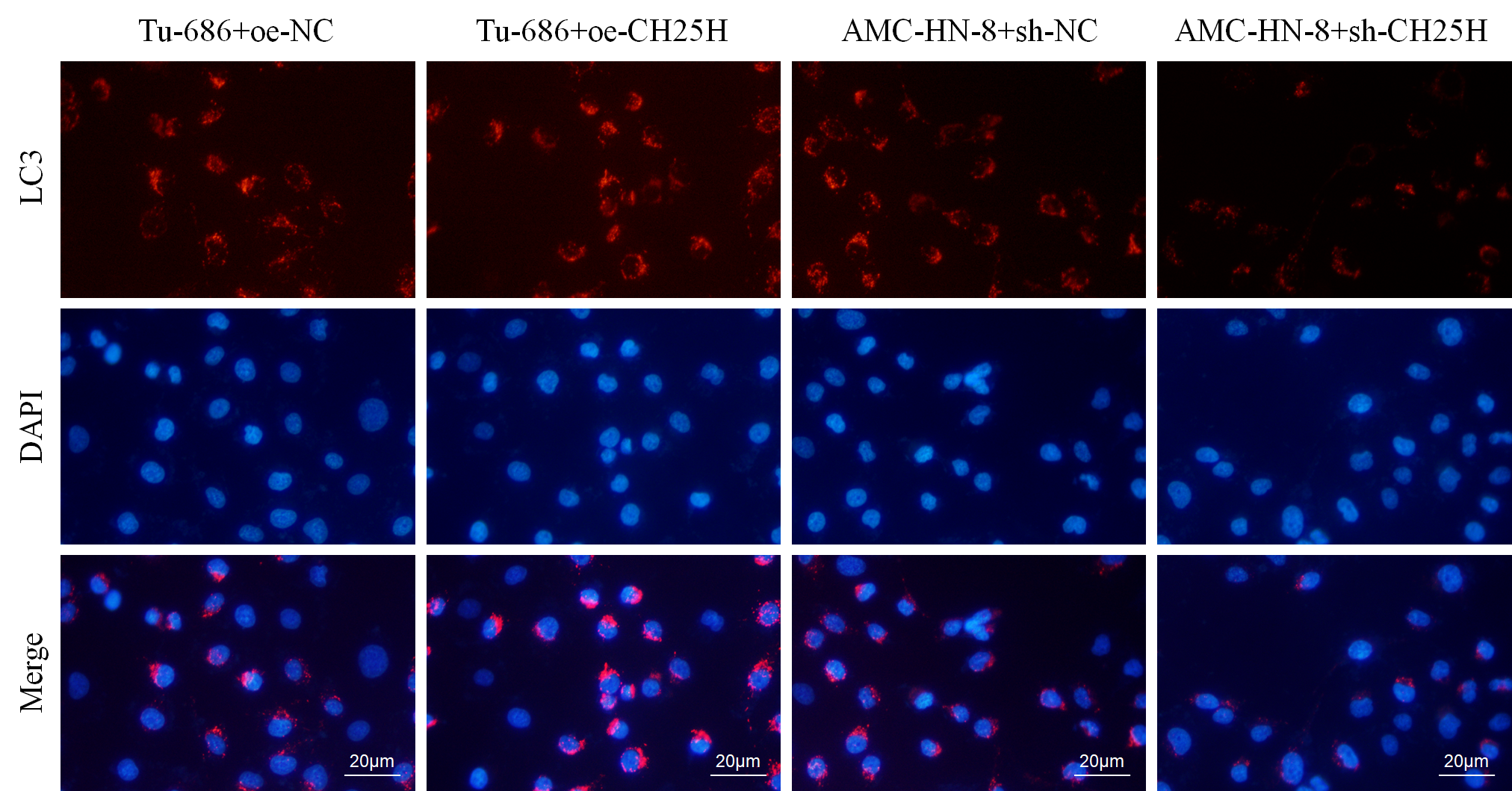

Supplement: Supplementary file 1 — Figure S1. After overexpressing CH25H in TU‐686 and knocking down CH25H in AMC‐HN‐8, the localization of autophagy‐related proteins was detected by immunofluorescence. [file CAM4-13-e70312-s006.tif]

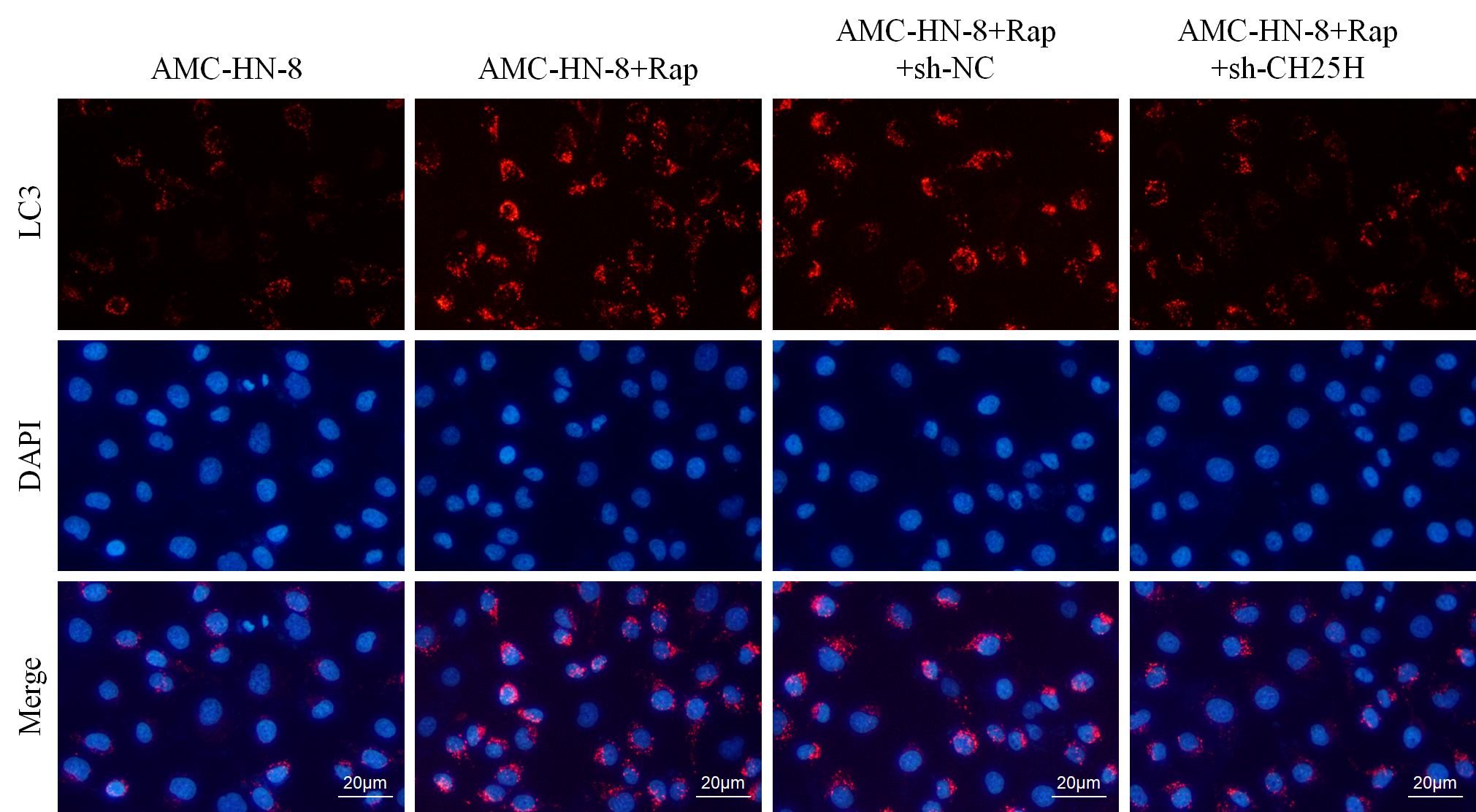

Supplement: Supplementary file 2 — Figure S2. After treating cells with autophagy agonists, the localization of autophagy‐related proteins was detected by immunofluorescence. [file CAM4-13-e70312-s003.tif]

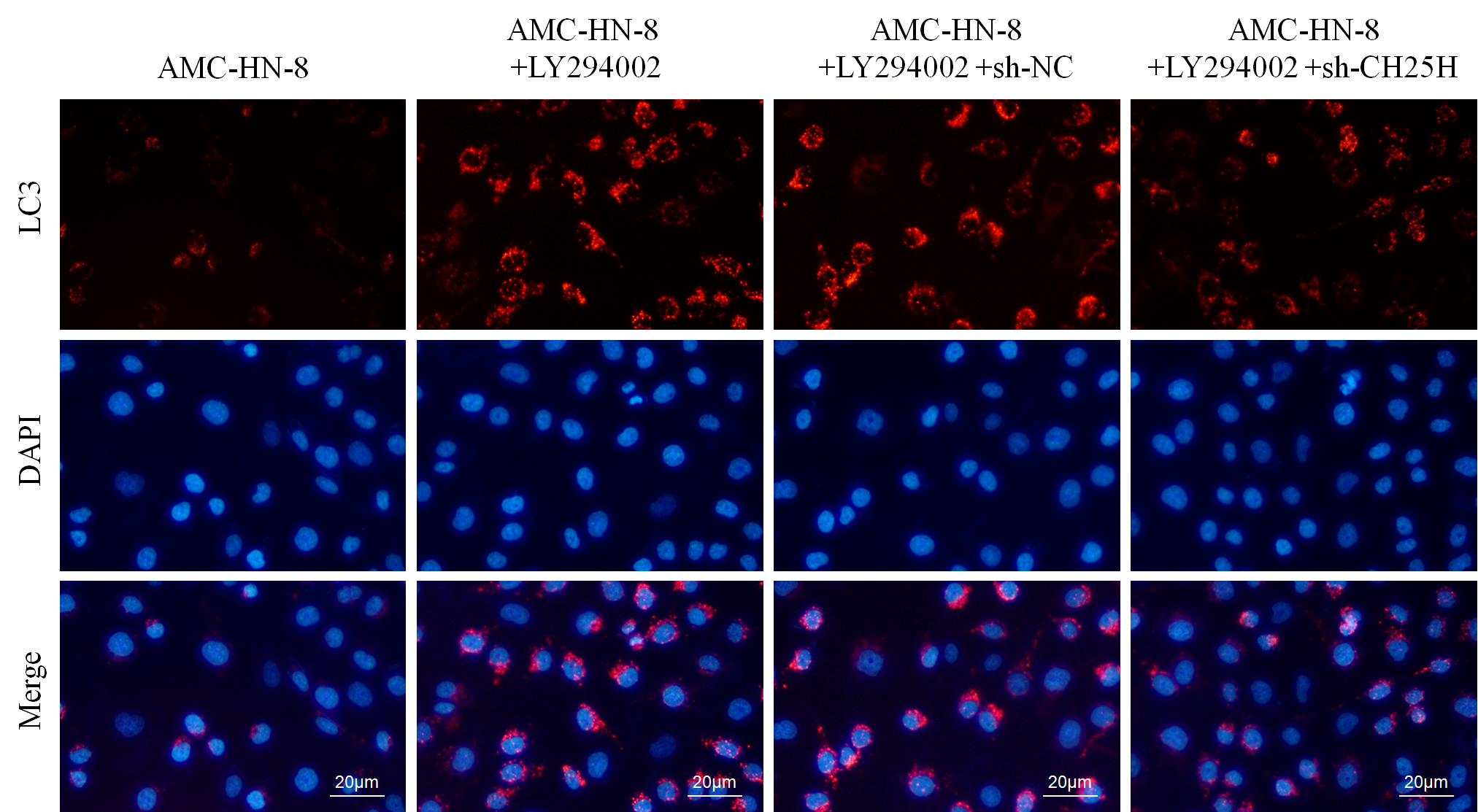

Supplement: Supplementary file 3 — Figure S3. After treating cells with LY294002, the localization of autophagy‐related proteins was detected by immunofluorescence. [file CAM4-13-e70312-s004.tif]

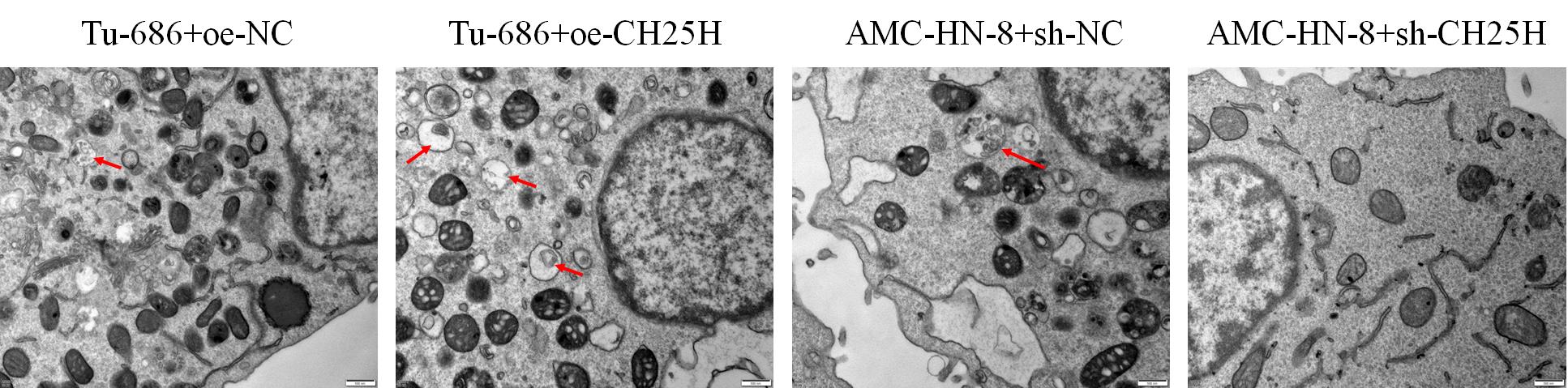

Supplement: Supplementary file 4 — Figure S4. After overexpressing CH25H in TU‐686 and knocking down CH25H in AMC‐HN‐8, the localization of autophagy‐related proteins was detected by electron microscope. [file CAM4-13-e70312-s005.jpg]

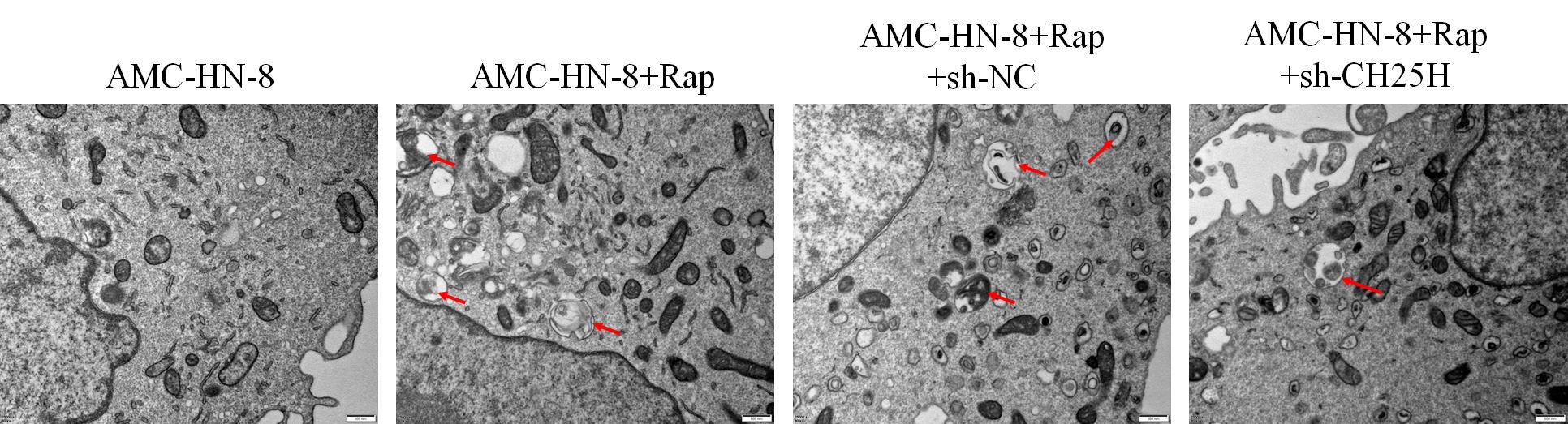

Supplement: Supplementary file 5 — Figure S5. After treating cells with autophagy agonists, the localization of autophagy‐related proteins was detected by electron microscope. [file CAM4-13-e70312-s001.jpg]

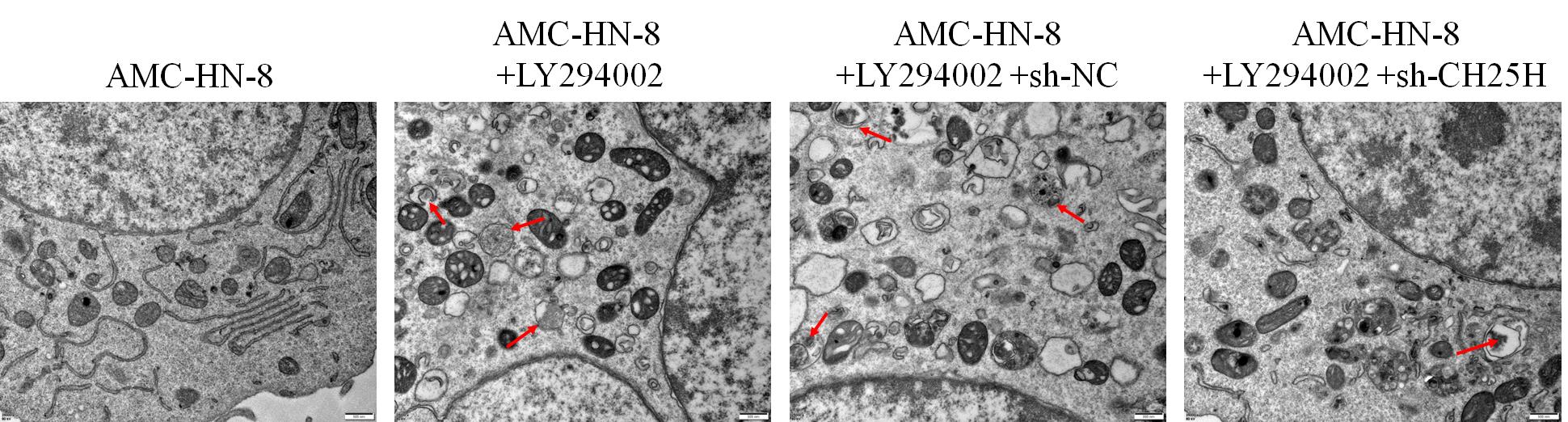

Supplement: Supplementary file 6 — Figure S6. After treating cells with LY294002, the localization of autophagy‐related proteins was detected by electron microscope. [file CAM4-13-e70312-s002.jpg]
